# Mandibular reconstruction with a ready-made type and a custom-made type titanium mesh after mandibular resection in patients with oral cancer

**DOI:** 10.1186/s40902-018-0175-z

**Published:** 2018-11-25

**Authors:** Won-bum Lee, Won-hyuk Choi, Hyeong-geun Lee, Na-rae Choi, Dae-seok Hwang, Uk-kyu Kim

**Affiliations:** Department of Oral and Maxillofacial Surgery, School of Dentistry, Pusan National University, 20, Geumo-ro, Mulgeum-eup, Yangsan, Gyeongsangnam-do South Korea

**Keywords:** Mandibular reconstruction, Squamous cell carcinoma, Titanium, CAD-CAM (computer-aided design and computer-aided manufacturing)

## Abstract

**Background:**

After the resection at the mandibular site involving oral cancer, free vascularized fibular graft, a type of vascularized autograft, is often used for the mandibular reconstruction. Titanium mesh (T-mesh) and particulate cancellous bone and marrow (PCBM), however, a type of non-vascularized autograft, can also be used for the reconstruction. With the T-mesh applied even in the chin and angle areas, an aesthetic contour with adequate strength and stable fixation can be achieved, and the pores of the mesh will allow the rapid revascularization of the bone graft site. Especially, this technique does not require microvascular training; as such, the surgery time can be shortened. This advantage allows older patients to undergo the reconstructive surgery.

**Case presentation:**

Reported in this article are two cases of mandibular reconstruction using the ready-made type and custom-made type T-mesh, respectively, after mandibular resection. We had operated double blind peer-review process. A 79-year-old female patient visited the authors’ clinic with gingival swelling and pain on the left mandibular region. After wide excision and segmental mandibulectomy, a pectoralis major myocutaneous flap was used to cover the intraoral defect. Fourteen months postoperatively, reconstruction using a ready-made type T-mesh (Striker-Leibinger, Freibrug, Germany) and iliac PCBM was done to repair the mandible left body defect.

Another 62-year-old female patient visited the authors’ clinic with pain on the right mandibular region. After wide excision and segmental mandibulectomy on the mandibular squamous cell carcinoma (SCC), reconstruction was done with a reconstruction plate and a right fibula free flap. Sixteen months postoperatively, reconstruction using a custom-made type T-mesh and iliac PCBM was done to repair the mandibular defect after the failure of the fibula free flap. The CAD-CAM T-mesh was made prior to the operation.

**Conclusions:**

In both cases, sufficient new-bone formation was observed in terms of volume and strength. In the CAD-CAM custom-made type T-mesh case, especially, it was much easier to fix screws onto the adjacent mandible, and after the removal of the mesh, the appearance of both patients improved, and the neo-mandibular body showed adequate bony volume for implant or prosthetic restoration.

## Background

After resection at the mandibular site involving oral cancer, free vascularized fibular graft, a type of vascularized autograft, is often used for the mandibular reconstruction. Also, titanium mesh (T-mesh) and particulate cancellous bone and marrow (PCBM), a type of non-vascularized autograft, can be used for the reconstruction.

Boyne successfully demonstrated the PCBM graft using T-mesh [1]. The main advantage of the T-mesh trays is the ability to maintain the spatial connection of the mandibular segments without the microvascular technique. With the T-mesh applied even in the chin and angle areas, an aesthetic contour with adequate strength and stable fixation can be achieved [16, 17], and the perforations of the mesh will allow the rapid revascularization of the bone graft site. Especially, this technique does not require microvascular training; therefore, the surgery time can be shortened. This advantage allows older patients to undergo the reconstructive surgery. This tray has adequate strength and many holes allowing the blood vessels to grow into bone. Also, the bone regeneration technique with “recombinant human bone morphogenetic protein in an acellular collagen sponge (rhBMP-2/ACS) method” was recently integrated in this reconstruction technique [2]. The purpose of this study was to review two cases of mandibular reconstruction using ready-made type and custom-made type T-mesh after mandibular resection, respectively.

## Case presentation

### Ready-made type T-mesh case

**Fig. 1:**
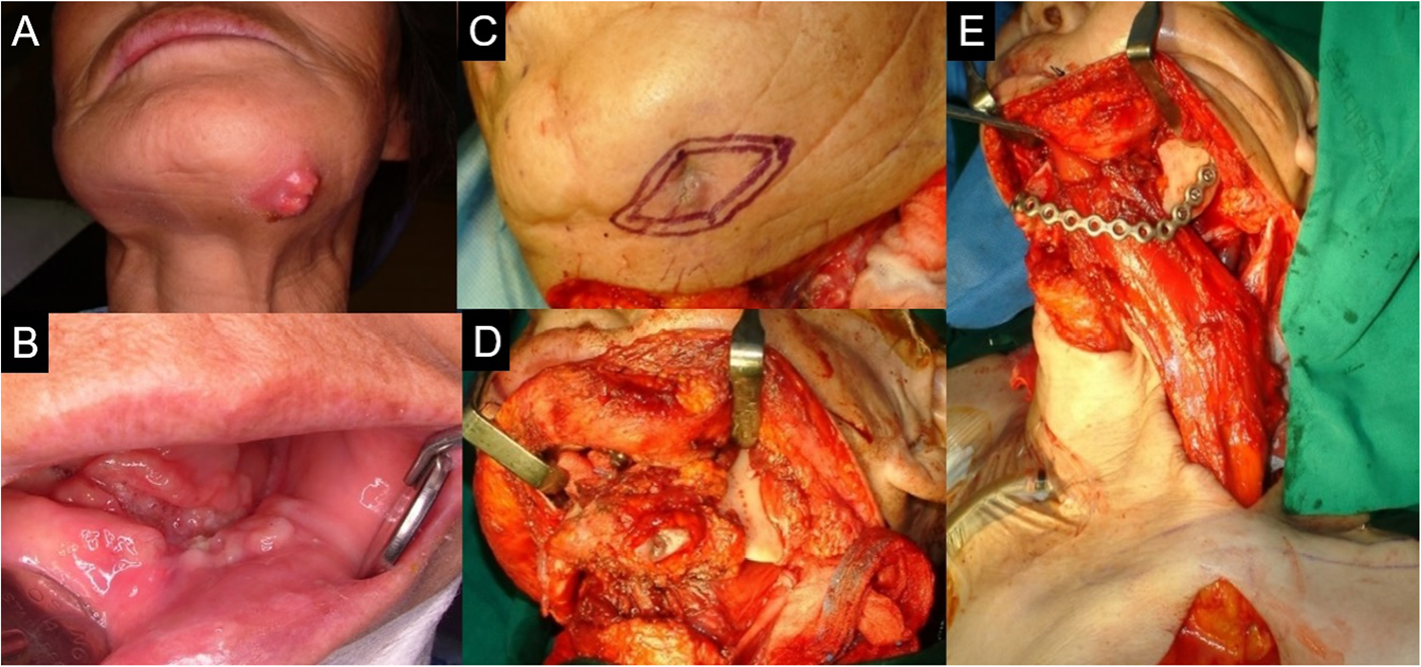
**a**, **b** Lesion on extraoral and intraoral site. **c**, **d** Extraoral excision outline and postoperative mandible state. **e** PMMC flap transfer to the resected mandible site

A 79-year-old female patient visited the authors’ clinic with gingival swelling and pain on the left mandibular region. She was in a mandible edentulous state. The lesion range was from the alveolar crest on the mandible left to the floor of the mouth. Incisional biopsy was done. The biopsy result indicated the presence of squamous cell carcinoma (cT4aN2cMx). After incisional biopsy, the patient was given neoadjuvant chemotherapy (Doxetaxel) for 1 day. Modified radical neck dissection (mRND) was done on the left side. On the right side, supraomohyoid neck dissection (SOHND) was done. Wide excision and segmental mandibulectomy was also done. Reconstruction was done with a reconstruction plate. Instead of free tissue transfer, a pectoralis major myocutaneous flap was used to cover the intraoral defect due to the patient’s poor general condition. The biopsy result indicated the presence of squamous cell carcinoma (pT4N2cMx). The patient was given postoperative radiotherapy 25 times (45 Gy) (Fig. 1).

**Fig. 2:**
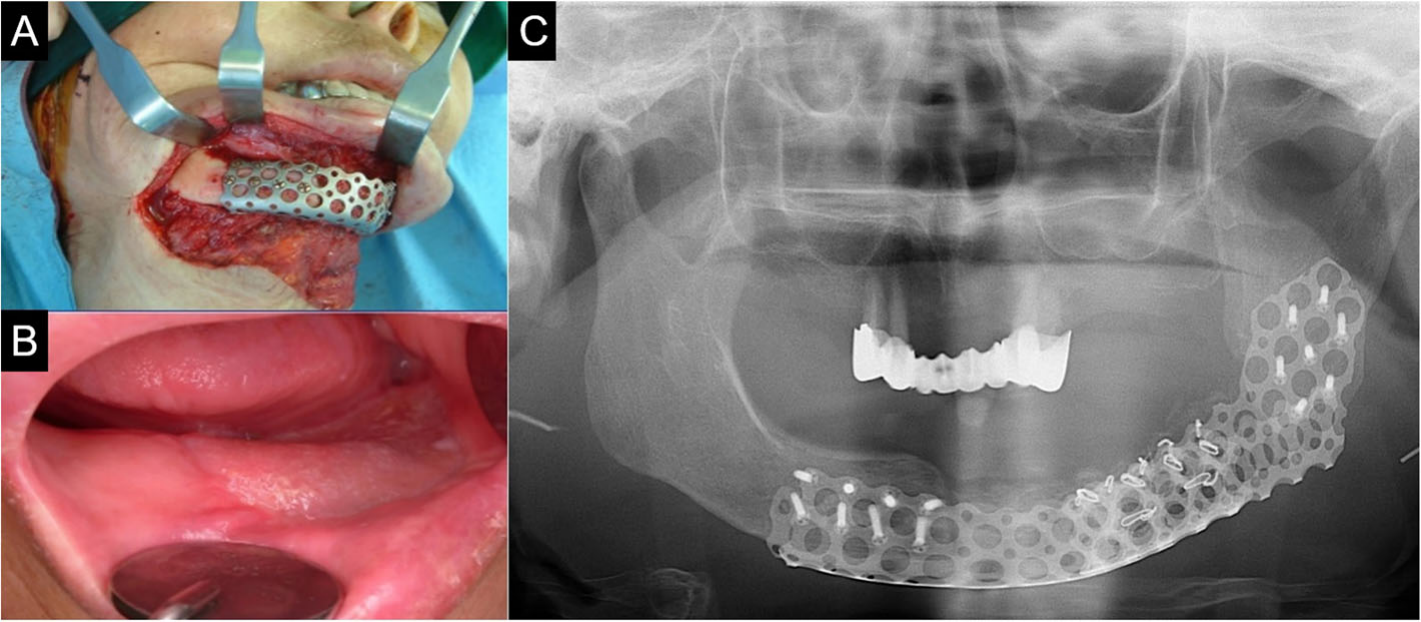
**a** Ready-made type T-mesh with iliac PCBM. **b** Healed PMMC skin flap on intraoral site. **c** Panoramic view on reconstructed mandible with T-mesh

Fourteen months postoperatively, reconstruction using ready-made type T-mesh (Striker-Leibinger, Freibrug, Germany) and iliac PCBM was done to repair the mandible left body defect (7 × 2.5 × 1.0 cm). After the reconstruction plate removal, corticocancelous block bone harvest was performed, including the iliac crest, and the Ti-mesh tray was filled with iliac PCBM. After that, the tray was adapted to the mandible and fixated using eight screws onto the anterior area, and seven screws onto the ramus area (Fig. 2)**.**

The patient did not come back for follow-up for 2 years and 3 months. Seven years and 6 months postoperatively, the patient revisited with orocutaneous fistula on the left chin, but neither intraoral fistula nor pus discharge was observed. Fistulectomy on the chin area was done at first to cover the T-mesh.

**Fig. 3:**
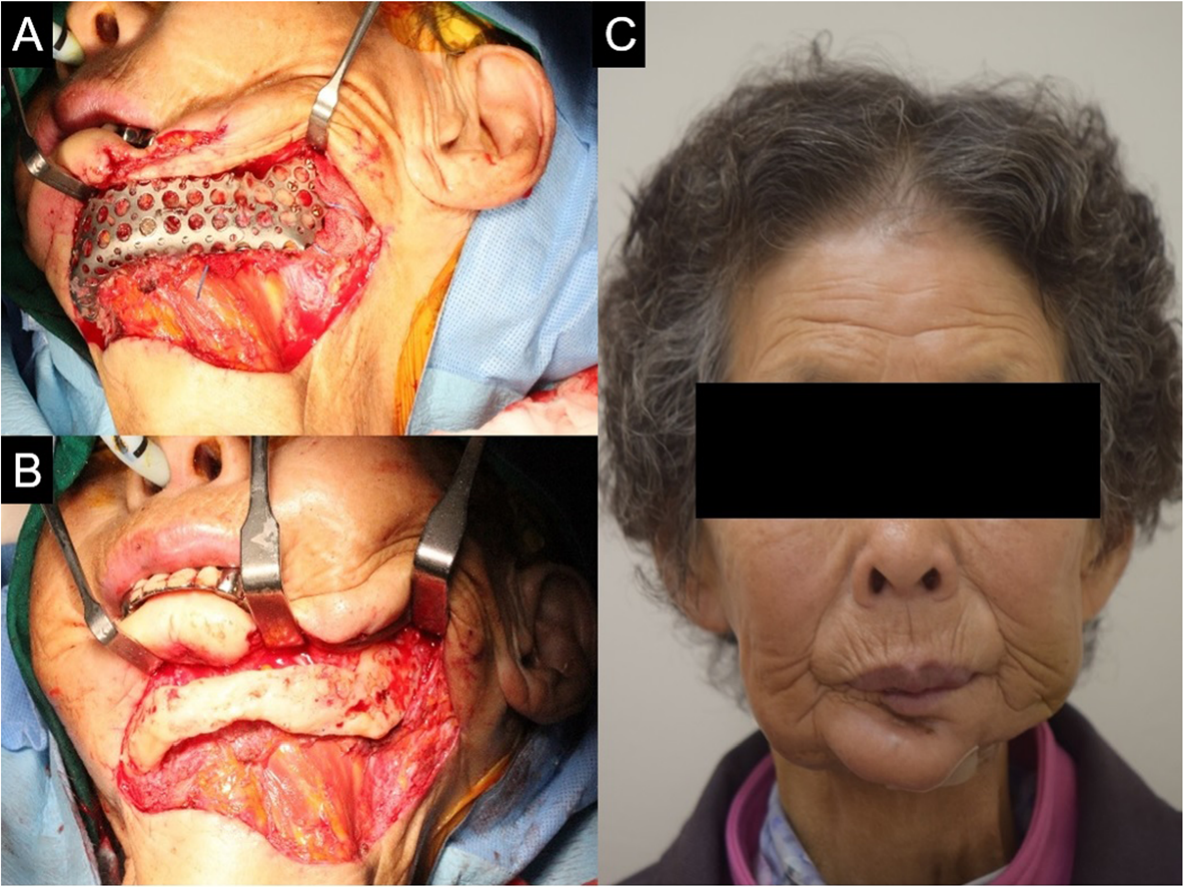
**a**, **b** Neomandible state before and after T-mesh removal. **c** The patient’s frontal view with neomandible

**Fig. 4:**
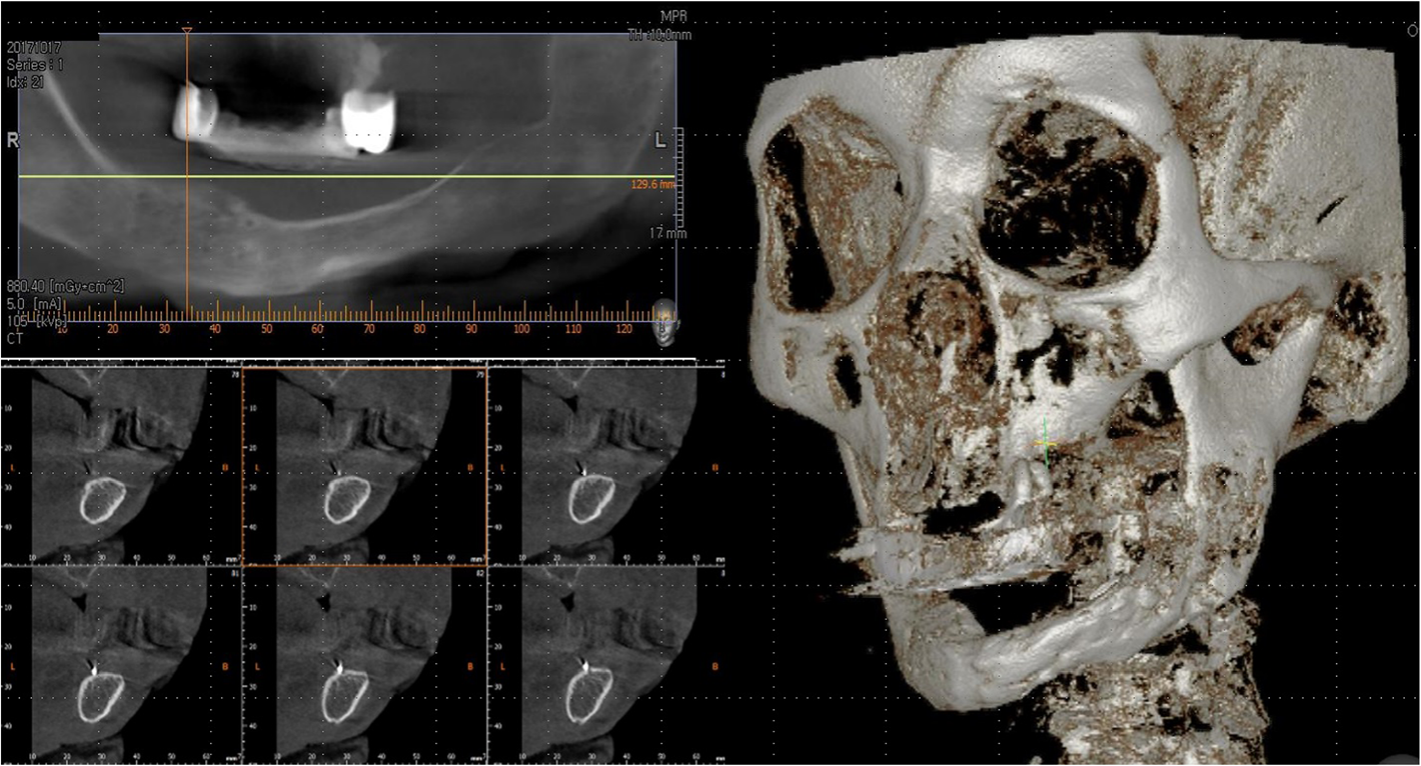
Neomandible body on CT view

Eight years and 8 months after the mandible reconstruction, there was T-mesh exposure on the left mandible area. As such, a Ti-mesh removal operation was scheduled. Eight years postoperatively (after the reconstruction surgery using T-mesh), the Ti-mesh tray and Leibinger screws (15 ea) were completely removed. The reconstruction site was found intact, and no inflammation was observed. Since after the Ti-mesh removal, no inflammation and complication have been observed. Also, an aesthetic contour of the mandible, with adequate functions, was achieved (Figs. 3 and 4).

### Custom-made type T-mesh case

**Fig. 5:**
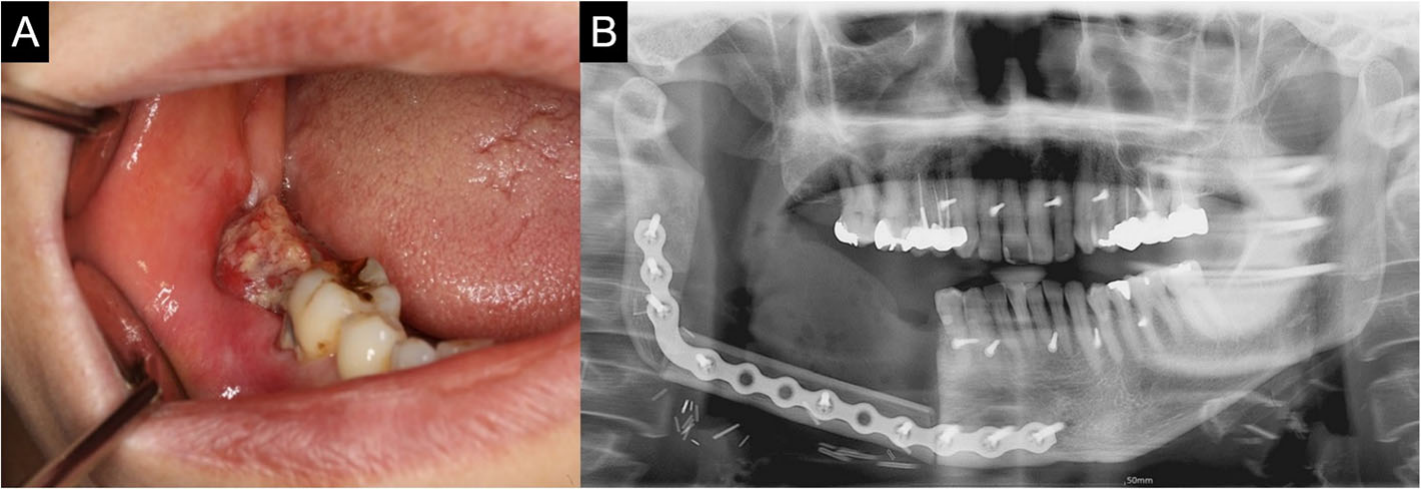
**a** Lesion on intraoral site. **b** Reconstruction with fibular free flap after mandible resection

**Fig. 6:**
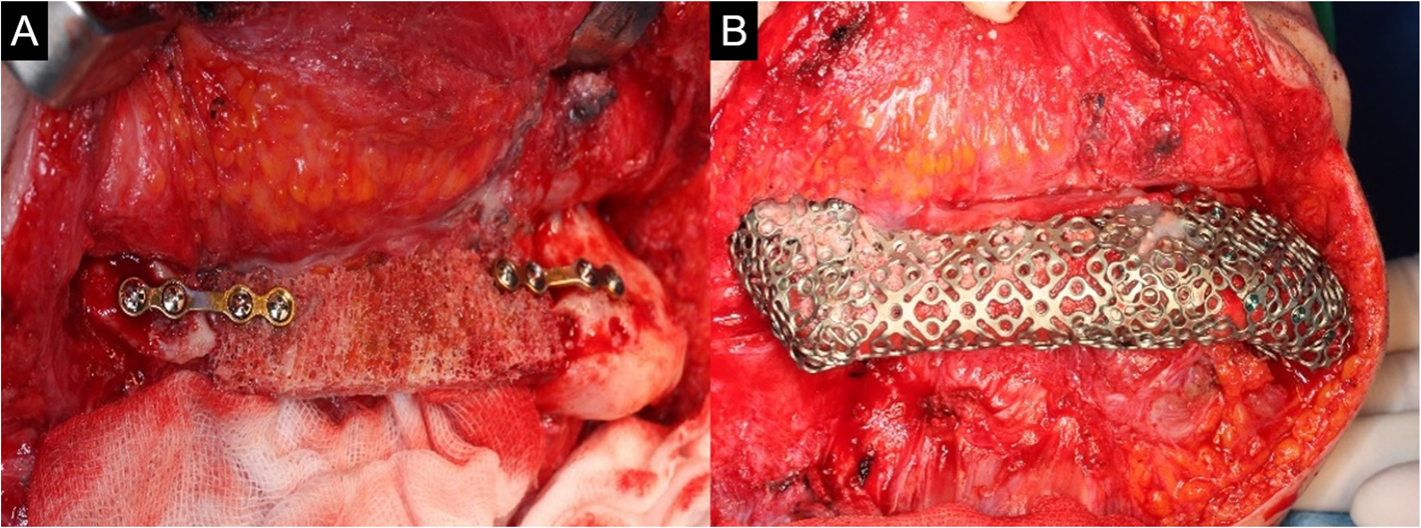
**a.** Intraoperative mandible with iliac block, PCBM **b**. Intraoperative mandible with  custom-made type T-mesh

**Fig. 7:**
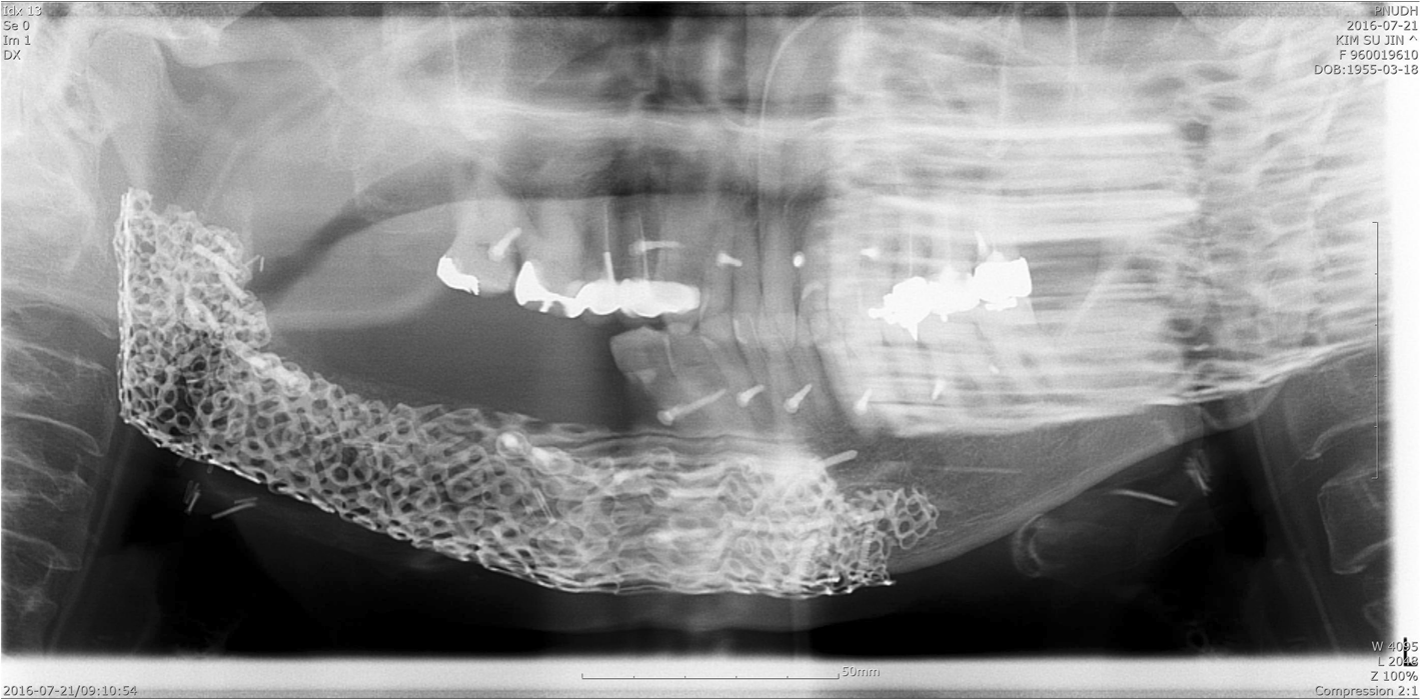
Panoramic view after Ti-mesh op

A 62-year-old female patient visited the authors’ clinic with pain on the right mandibular region. Incisional biopsy was done. The biopsy result indicated the presence of squamous cell carcinoma (cT4aN0M0). After incisional biopsy (20 days later), SOHND was done, along with wide excision and segmental mandibulectomy. Reconstruction was also done with a reconstruction plate and a right fibula free flap (Fig. 5). The biopsy result indicated the presence of squamous cell carcinoma (pT4aN0M0). One month postoperatively, the intraoral fibular skin flap was infected due to the emergency care that was given to the patient (due to a cardiologic problem that arose after the operation). The infected fibular segment was removed 1 month postoperatively. One year postoperatively, fibular bone exposure was observed. The two screws on the mandible posterior area, which had been used for fibular bone fixation, were removed. Complete curettage was done on the infected fibular bone. Although no wound dehiscence was observed after the curettage for 1 year and 4 months, reconstruction was done using custom-made type T-mesh and iliac PCBM and platelet-rich plasma (PRP), which has growth factors for bone healing enhancement [6], to repair the mandibular defect. The CAD-CAM T-mesh was made prior to the operation, at the laboratory. After the reconstruction plate removal, the iliac crest including a 7 × 5 cm block bone and PCBM was harvested and fixated using two mini-plates. The T-mesh tray was then adapted to the mandible, and the T-mesh tray was filled with an additional particulate iliac bone (Figs. 6 and 7).

**Fig. 8:**
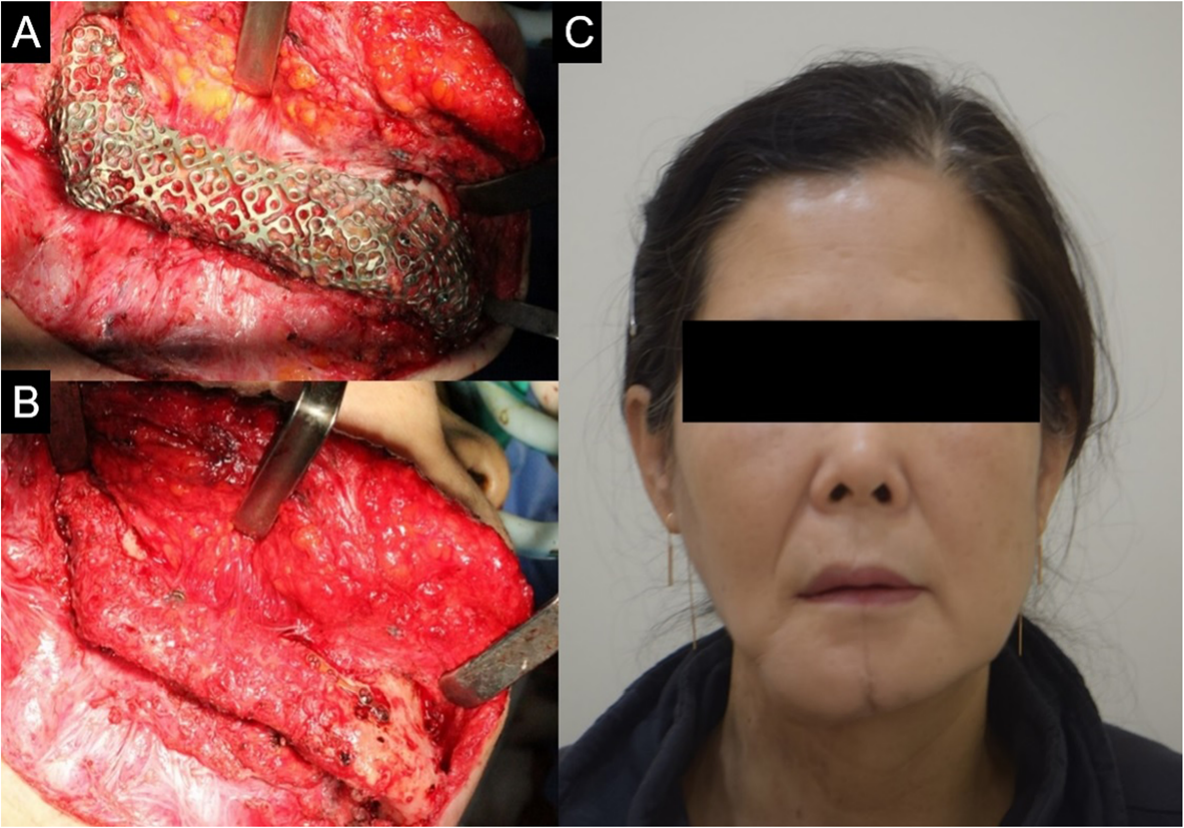
**a**, **b** Neomandible state before and after T-mesh removal. **c** The patient’s frontal view with neomandible

**Fig. 9:**
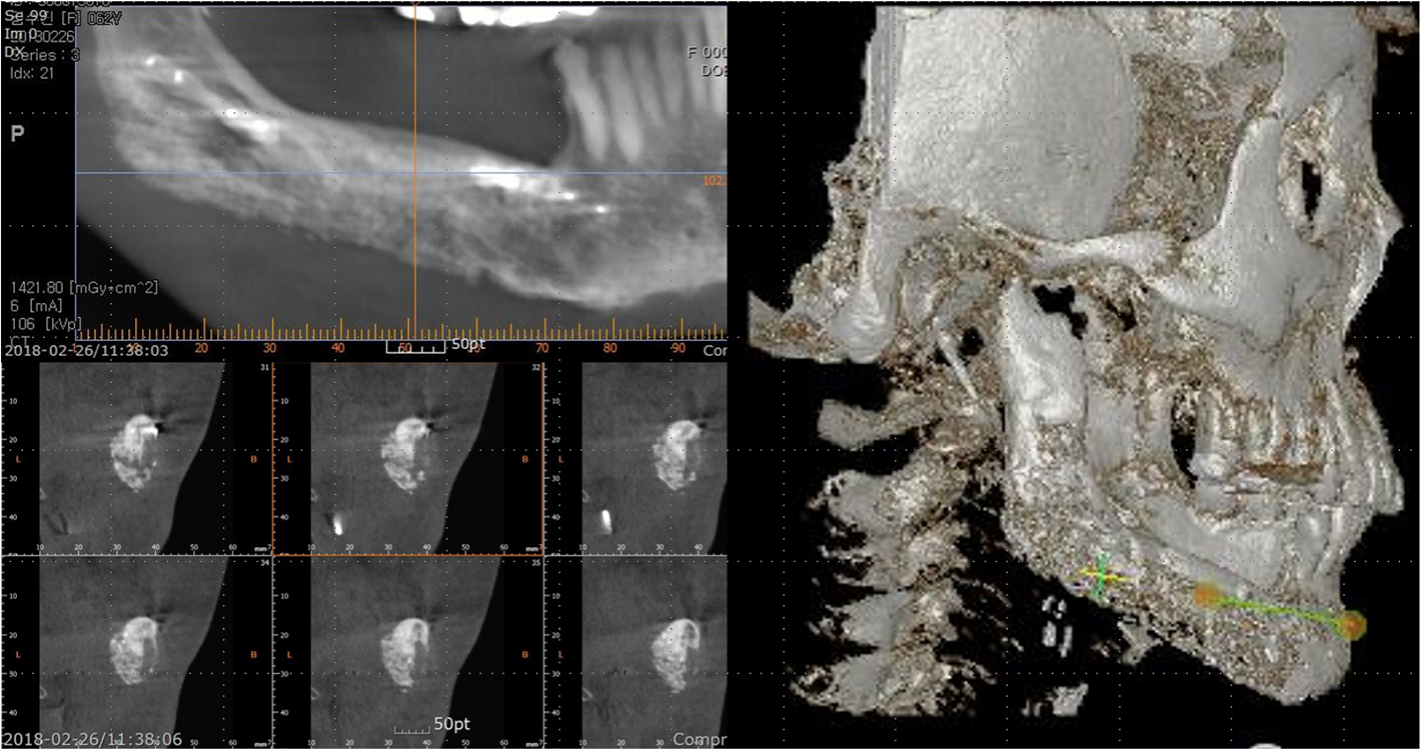
Neomandible body on CT view

One year and 6 months postoperatively (after the reconstruction surgery), no fistula and swelling were observed. The T-mesh tray (except the mini-plate) was completely removed. The reconstruction site was found intact, and no inflammation was observed. Since after the T-mesh removal, no inflammation and complication have been observed (Figs. 8 and 9).

## Discussion

After the mandibular resection, major reconstruction is done, and for this, two methods are currently in use: microvascular bone flap transfer and PCBM graft with a T-mesh tray [3]. Mowlem [4] introduced the superior osteogenic potential of cancellous bone grafts in 1944. The principle of PCBM was explained by the Axhausen [5] theory of osteogenesis, which explained that the surviving transplanted cells proliferate and form new random osteoids. Cancellous bone has the advantage of allowing resistance against infection, maximum survival, fast vascular ingrowth, and early incorporation into the host bone and the surrounding soft tissues. Theoretically, platelets are known to release growth factors. Marx et al. reported evidence of a 1.62- to 2.16-fold greater radiographic maturation rate in grafts with PRP compared to grafts without PRP [6]. Also, a new-bone reconstruction technique using recombinant human bone morphogenetic protein-2 (rhBMP-2) without concomitant autogenous bone graft was introduced [7]. PRP was also used when grafting the iliac PCBM and block bone with the custom-made type T-mesh.

In our second case, we also used PRP technique in iliac PCBM for expecting role of growth factors including BMP in PRP to stimulate new bone formation.

Dumbach et al. [8] reported the T-mesh (Dumbach Titan Mesh System; Striker-Leibinger, Freibrug, Germany) for mandibular reconstruction in 1987. In both patients with the T-mesh applied even in the chin and angle areas, an aesthetic contour with adequate strength and stable fixation can be achieved. This advantage presents a great opportunity for implant prosthesis restoration. Although vascularized bone grafts have little bone and suboptimal contour bulk, nonvascularized bone grafts can provide a better bone bulk in which to place the implants [8]. Carlson et al. have shown the total bulk of the fibula to be only 26% of the normal mandible [3]. The pores of the mesh allow rapid revascularization of the bone graft site. Especially, this technique does not require microvascular training; therefore, the surgery time can be shortened. This advantage allows older patients to undergo the reconstructive surgery, but especially in patients with osteoporosis, the resorption rate of the bone graft may be higher.

In our present cases, two cases showed good bony healing in iliac PCBM for formation of neomandible via revascularization.

The current study indicated that there are criteria in many cases [9]. If the patient has had preoperative radiation therapy, if the defect is large, or if immediate soft-tissue transfer is also required, transfer of vascularized tissue is indicated. After radiation therapy, most of the osteocytes in the graft will die, which will increase the chances of infection. This problem may be solved with microsurgical techniques and free tissue transfers [10]. Nonvascularized bone grafts are most successful in non-irradiated patients who have adequate soft tissue, and where the defect is shorter (< 6 cm). Marx et al. [11] stated that the greatest challenge has been seen in patients irradiated with > 50 Gy. He also suggested that the success rate for nonvascularized grafts placed in irradiated tissue (> 60–65 Gy) can be improved by the use of hyperbaric oxygen preoperatively [11]. The patient (the ready-made type T-mesh case), however, was given neoadjuvant chemotherapy (Doxetaxel) for 1 day after incisional biopsy, and 25-time postoperative radiotherapy (45 Gy). No wound dehiscence or infection was found during the follow-up period.

In our first case, she received postoperative radiation (45 Gy) before bone grafting, but the graft of PCBM with ready-made type T-mesh was successfully healed.

Lino et al. [12] stated that the precise fitting and shaping of ready-made type T-mesh (Dumbach Titan Mesh System; Striker-Leibinger, Freibrug, Germany) is sometimes difficult and time-consuming. Besides, the Dumbach Titan Mesh System is no longer commercially available. To solve this problem, the preoperative preparation of custom-made type T-mesh with the use of a three-dimensional (3D) model was recently reported by Goto et al. [13] and Yamada et al. [14] Based on the preoperative computed tomography data, the defects are reconstructed using the mirror image of the non-defect side. The T-mesh produced using a 3D skull model can reproduce the natural configuration on the mandible [15].

In our second case, the patient’s custom-made type T-mesh was made with 3D mandible via CAD-CAM technique and secured accurately on her mandibular defect area.

## Conclusion

In both reported cases of mandibular reconstruction with iliac particulate cancellous bone and marrow (PCBM) and titanium mesh (T-mesh), sufficient new-bone formation was observed in terms of volume and strength. This technique has enabled highly aesthetic and functional results to be obtained. It is suggested as a treatment of choice for mandibular reconstruction T-mesh and iliac PCBM; however, only on selected mandibular bony defect patients.

## Abbreviations

PCBM: Particulate cancellous bone and marrow
PRP: Plate-rich plasma
rhBMP-2: Recombinant human bone morphogenetic protein-2
rhBMP-2/ACS: Recombinant human bone morphogenetic protein-2 in an acellular collagen sponge
T-mesh: Titanium mesh

## References

[1] Boyne PJ (1969). Restoration of osseous defects in maxillofacial casualties. J Am Dent Assoc.

[2] Goh BT, Lee S, Tideman H, Stoelinga PJ (2008). Mandibular reconstruction in adults: a review. Int J Oral Maxillofac Surg.

[3] Lee SC (1996). Mandibular reconstruction with titanium mesh tray and autogenous particulated cancellous bone and marrow. J Korean Assoc Oral Maxillofac Surg.

[4] Bell RB, Andersen P, Fernandes R (2018) Oral, head and neck oncology and reconstructive surgery, 1st edn. Elsevier Health Science:208–220

[5] Marx RE, Carlson ER, Eichstaedt RM (1998). Platelet-rich plasma: growth factor enhancement for bone grafts. Oral Surg Oral Med Oral Pathol Radiol Endod.

[6] Carlson ER, Marx RE (1996). Mandibular reconstruction using cancellous bone grafts. J Oral Maxillofac Surg.

[7] Mowlem R (1944). Report of eighty-five cancellous chip grafts. Lancet.

[8] Axhausen W (1956). The osteogenic phase of regeneration of bone: a historical and experimental study. J Bone Joint Surg.

[9] Cicciu M, Herford AS, Cicciu D, Tandon R, Maiorana C (2014). Recombinant human bone morphogenetic protein-2 promote and stabilize hard and soft tissue healing for large mandibular new bone reconstruction defects. J Craniofac Surg.

[10] Dumbach J, Rodemer H, Spitzer WJ, Steinhauser EW (1994). Mandibular reconstruction with cancellous bone, hydroxylapatite and titanium mesh. J Craniomaxillofac Surg.

[11] Pogrel MA, Podlesh S, Anthony JP, Alexander J (1997). A comparison of vascularized and nonvascularized bone grafts for reconstruction of mandibular continuity defects. J Oral Maxillofac Surg.

[12] Riedinger D (1990). Knochenersatz einschließlich Mikrogefaßchirurgie und Rehabilitation mit Implantaten.

[13] Marx RE, Ames JR (1982). The use of hyperbaric oxygen therapy in bony reconstruction of the irradiated and tissue deficient patient. J Oral Maxillofac Surg.

[14] Lino M, Fukuda M, Nagai H, Hamada Y, Yamada H, Nakoka K et al.(2009) Evaluation of 15 mandibular reconstructions with Dumbach Titan Mesh-System and particulate cancellous bone and marrow harvested from bilateral posterior ilia;107:e1-e8 10.1016/j.tripleo.2008.12.018 19201629

[15] Goto M, Katsuki T. Noguchi N, Hino N(1997) Surgical simulation for reconstruction of mandibular bone defects using photocurable plastic skull models: report of three cases;55:772–780 10.1016/s0278-2391(97)90597-8 9216515

[16] Yamada H, Nakaoka K, Sonoyama T, Kumagai K, Ikawa T, Shigeta Y (2016). Clinical usefulness of mandibular reconstruction using custom-made titanim mesh tray and autogenous particulate cancellous bone and marrow harvested from tibia and/or illa. J Cranio Surg.

[17] Takato T, Mori Y, Fujihara Y, Asawa Y, Nishizawa S, Kanazawa S (2014). Preclinical and clinical research on bone and cartilage regenerative medicine in oral and maxillofacial region. Oral science international.

